# Rebound of Respiratory Virus Activity and Seasonality to Pre‐Pandemic Patterns

**DOI:** 10.1002/jmv.70658

**Published:** 2025-10-23

**Authors:** Rainer Gosert, Klaudia Naegele, Marvin Weiss, Roland Bingisser, Christian H. Nickel, Jakob Meyer, Martin Siegemund, Stefano Bassetti, Sarah Dräger, Christoph T. Berger, Fabian Franzek, Ulrich Heininger, Julia Bielicki, Hans H. Hirsch, Peter M. Keller, Maja Weisser, Nina Khanna, Sarah Tschudin‐Sutter, Karoline Leuzinger

**Affiliations:** ^1^ Clinical Virology University Hospital Basel Basel Switzerland; ^2^ Infectious Diseases & Hospital Epidemiology University Hospital Basel; ^3^ Emergency Medicine University Hospital Basel Basel Switzerland; ^4^ Intensive Care Unit University Hospital Basel Basel Switzerland; ^5^ Internal Medicine University Hospital Basel Basel Switzerland; ^6^ University Center for Immunology University Hospital Basel Basel Switzerland; ^7^ Research and Analytics, Department of Informatics University Hospital Basel Basel Switzerland; ^8^ Infectious Diseases & Hospital Epidemiology University Medical Center Basel‐Land Liestal Switzerland; ^9^ Pediatric Infectious Diseases & Hospital Epidemiology University Children Hospital Basel Basel Switzerland; ^10^ Transplantation & Clinical Virology, Department Biomedicine University of Basel Basel Switzerland; ^11^ Clinical Bacteriology and Mycology, Laboratory Medicine University Hospital Basel Basel Switzerland

**Keywords:** atypical bacteria, CARV, community‐acquired respiratory virus, COVID‐19, influenza, multiplex nucleic acid testing, respiratory syncytial virus, RSV, SARS‐CoV‐2, syndromic testing

## Abstract

The emergence of SARS‐CoV‐2 and the implementation of non‐pharmaceutical interventions (NPIs) profoundly disrupted the transmission dynamics of respiratory viruses, altering their epidemiology and seasonality. However, comprehensive long‐term data on these shifts and their post‐pandemic implications remain limited. This study analyzed syndromic multiplex panel testing data from 83′823 respiratory specimens collected from 56,519 patients with respiratory tract infections (RTIs) at two tertiary care centers in northwestern Switzerland to systematically assess changes in respiratory virus circulation, seasonality, age distribution, and disease burden across pre‐pandemic (2010–2019), pandemic (2019–2022), and post‐pandemic (2022–2024) periods. Pre‐pandemic, influenza virus (IV), respiratory syncytial virus (RSV), human coronavirus (HCoV), human metapneumovirus (hMPV), and human parainfluenza virus (HPIV) followed distinct seasonal patterns. During the pandemic, SARS‐CoV‐2 replaced these viruses, leading to a 70–90% decline in their activity (*p* < 0.001), while rhinovirus/enterovirus and adenovirus were less affected. After NPIs were lifted, substantial off‐season activity with markedly higher case numbers and more hospitalizations, especially among pediatric patients, occurred for IV‐A/B, RSV, and atypical bacteria. In post pandemic years, virus‐specific seasonality is rebounding, with patterns resembling those seen pre‐pandemic. However, higher case numbers, increased hospitalizations, and sustained shifts in age distribution persist. The COVID‐19 panemic significantly impacted the etiology, seasonality, and age distribution of RTIs. As NPIs were eased, susceptibility to RTIs, particularly among pediatric patients, increased, resulting in more hospitalizations. While post‐pandemic periods show a return to pre‐pandemic activity patterns, ongoing monitoring is essential to anticipate shifts in respiratory virus dynamics as immunity levels and virus characteristics evolve.

## 1 Introduction

Respiratory tract infections (RTIs) are a leading cause of morbidity and mortality, especially in vulnerable populations including immunocompromised patients, young children and older persons. These infections are characterized by an epidemic occurrence with virus‐specific seasonality [1]. Climatic factors such as temperature and humidity affect respiratory virus stability and transmission rates, and may affect the host′s intrinsic, innate, and adaptive immune responses to RTIs, thereby contributing to increased viral activity during fall and winter [2]. The onset of the COVID‐19 pandemic in early 2020 coincided with the seasonal peaks of several respiratory viruses, including influenza virus A and B (IV‐A/B), respiratory syncytial virus type A and B (RSV), and seasonal human coronaviruses (HCoV) [3]. In response to the global COVID‐19 pandemic, governments worldwide implemented a range of non‐pharmaceutical interventions (NPIs), including lockdowns, travel restrictions, social distancing, mask mandates, and school and business closures, to mitigate SARS‐CoV‐2 transmission. While these measures effectively reduced the spread of SARS‐CoV‐2, they also significantly impacted the transmission dynamics of other respiratory viruses [3, 4, 5, 6, 7], raising concerns about persistent shifts in their epidemiology. However, data on changes in the spectrum of circulating viruses and atypical bacteria, along with their activity, seasonality, and associated disease burden compared to pre‐pandemic periods, remain limited. This study aims to elucidate the etiology, seasonality, age distribution, and associated disease burden of RTIs in patients from 2010 to 2024 at two tertiary care hospitals in northwestern Switzerland.

## 2 Materials and Methods

### 2.1 Patient Cohorts and Inclusion Criteria

The University Hospital Basel is the largest tertiary care facility in Northwestern Switzerland, with a capacity of 700 beds. The University Children′s Hospital Basel, with a capacity of 120 beds, is the leading healthcare provider for children and adolescents in northwestern Switzerland and serves as a specialized referral center. This retrospective study included patients who presented with RTI symptoms to the outpatient clinics or the adult and pediatric emergency departments of both hospitals and received syndromic multiplex panel testing between July 2010 and June 2024. Patients who were admitted following their initial presentation with acute RTI symptoms were included in the analysis. In contrast, hospital‐acquired infections, defined as patients who developed RTI symptoms after hospitalization are not included in this analysis. Both, the University Hospital Basel and the University Children′s Hospital Basel, perform among the highest numbers of syndromic multiplex panel tests for patients with RTIs in Switzerland [3, 8], providing comprehensive, high‐quality epidemiological data for assessing long‐term trends in respiratory pathogen dynamics.

### 2.2 Timeline of Implementation of Non‐Pharmaceutical Interventions

In Switzerland, governmental NPIs were introduced in calendar week 11 of 2020. The initial measures included canceling mass gatherings, closing schools and cultural venues. These physical distancing measures were later expanded by banning all gatherings and closing non‐essential business. Restrictions were cautiously lifted 7 weeks later but reinstated during the following winter season as Alpha, Delta and Omicron variants surged across Europe. NPIs began to ease in February 2022 and were fully lifted by April 2022 (Supplementary Table 1).

### 2.3 Syndromic Multiplex Panel Testing

Respiratory specimens were collected using two swabs from the nasopharyngeal and oropharyngeal sites, respectively, and combined into a single universal transport medium tube (Copan, Brescia, Italy). In younger children, only nasopharyngeal swabs were obtained.

All respiratory specimens were sent to the Clinical Virology Unit at the University Hospital Basel, which operates a dedicated emergency diagnostic service available 24 h a day, 7 days a week. Upon receipt, specimens were immediately processed in the diagnostic laboratory, and multiplex panel testing was initiated without delay [3, 9, 10, 11]. In practice, respiratory samples were analyzed within minutes to a maximum of 1 h after reciept, ensuring a time‐to‐result of ≤ 4 h from collection. This rapid workflow guaranteed optimal pre‐analytical conditions, robust diagnostic performance, and timely results to support clinical triage and infection control. Over the study period, three different syndromic multiplex panel testing systems were utilized, targeting both bacterial and viral pathogens. From July 2010 to January 2016, the RespiFinder‐22® Respiratory Pathogen Panel (RPP; PathoFinder, Maastricht, Netherlands) [10] was routinely used, which detects 18 viral and four bacterial respiratory pathogens. In January 2016, syndromic testing was switched to the NxTAG RPP on the MAGPIX platform (Diasorin, Saluggia, Italy) [10], which detects 18 viral and 2 bacterial pathogens. In November 2016, testing transitioned to the Biofire FilmArray RPP (bioMérieux, Marcy‐l′Étoile, France) [3], which detects 18 viral and four bacterial targets. Additionally, since October 2021, the Xpert® Xpress‐CoV‐2/Flu/RSV‐Plus system (Cepheid, CA, USA) [9] has been employed, targeting the three most critical viral respiratory pathogens in tertiary care settings. Since *Bordetella pertussis and Bordetella parapertussis* were only partially covered by RespiFinder‐22® RPP and NxTAG RPP, both bacterial pathogens were detected alongside the syndromic multiplex panel tests using laboratory‐developed tests targeting an insertion sequence [11] (Supplementary Table 2).

### 2.4 Statistics and Data Analysis

All statistical data analyses were performed in R (https://www.r-project.org/), and Prism (version 10; Graphpad Software, CA, USA) was used for data visualization.

For patients with repeated testing, results were consolidated into infection episodes to avoid double‐counting. Consecutive positive tests for the same respiratory pathogen within 90 days were classified as a single infection episode, acknowledging that viral shedding can persist for prolonged periods, particularly in immunocompromised individuals. Positive tests for the same respiratory pathogen occurring more than 90 days apart were considered new episodes. Detections of different respiratory pathogens were always treated as separate events regardless of timing. The 90‐day interval was chosen as it exceeds typical shedding durations in immunocompetent hosts while also accommodating prolonged persistence occasionally described in immunocompromised patients [12, 13, 14, 15].

In cases of co‐detections, each respiratory pathogen was counted independently in pathogen‐specific analyses (e.g., a sample positive for both RSV and HAdV contributed to both RSV and HAdV counts). For patient‐level outcomes such as unique case numbers or hospitalizations, however, coinfected patients were counted only once to avoid duplication.

Hospitalizations associated with IV‐A/B or RSV were defined as cases in which patients presented with acute RTI symptoms to the emergency or outpatient clinics, tested positive for the respective pathogen by syndromic multiplex panel testing, and were admitted to the hospital within 24 h. This definition was applied consistently across pandemic and post‐pandemic periods to allow a comparable assessment of virus‐associated hospitalization burden.

## 3 Results

A total of 83′823 respiratory clinical specimens were submitted for routine syndromic multiplex panel testing from 56,519 patients with RTIs (female: 26,382, 46.7%; pediatric patients ≤ 18 years: 13,187, 23.3%) between July 2010 and June 2024 (Supplementary Table 3). Median age of adult patients was 66 years (range: 19 to 111 years), and 6 years (range: 1 to 18 years) for pediatric patients.

**Figure 1:**
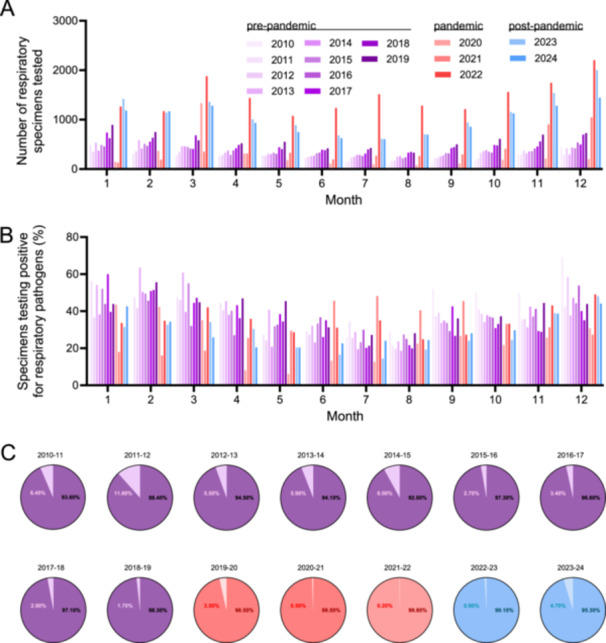
Surveillance results for respiratory viruses and atypical bacteria from 2010 to 2024. Number of respiratory clinical specimens (*n* = 83,823) and the percentage of positive tests for non‐SARS‐CoV‐2 respiratory viruses and atypical bacteria in patients with respiratory tract infections (*n* = 56,519). For non‐SARS‐CoV‐2 respiratory viruses and atypical bacteria included in the syndromic multiplex panel tests see Supplementary Table 2. Sampling years are color‐coded: shades of purple for pre‐pandemic, red for pandemic, and blue for post‐pandemic periods. (A) Number of respiratory clinical specimens tested by syndromic multiplex panel tests per month for the indicated year (total *n* = 83,823). (B) Percentage of specimens testing positive for any non‐SARS‐CoV‐2 respiratory virus or atypical bacterium among all respiratory clinical specimens tested by syndromic multiplex panel tests per month for the indicated year. (C) Percentage of non‐SARS‐CoV‐2 respiratory virus (represented in the dark shades of purple, red, and blue), and atypical bacterial detections (represented in light shades purple, red, and blue) among all respiratory clinical specimens tested positive by syndromic multiplex panel tests for the indicated season. Seasons are defined from July 1st to June 30th of the indicated years.

A median of 401 syndromic multiplex panel tests for non‐SARS‐CoV‐2 respiratory pathogens were conducted monthly, with a surge in March 2020, coinciding with the initial detection of SARS‐CoV‐2 in Switzerland. Although syndromic multiplex panel testing decreased after the pandemic, it remained higher than pre‐pandemic levels (Figure 1A). The overall test positivity rates for non‐SARS‐CoV‐2 respiratory pathogens were comparable across pre‐pandemic periods, ranging from 35.1% to 69.2%. These rates significantly decreased during the pandemic and rebounded in the post‐pandemic periods (Figure 1B). Bacterial pathogens like *M. pneumoniae* and *B. pertussis* were nearly absent in pandemic years but reemerged in the 2023–24 season, closely aligning with pre‐pandemic positivity rates (Figure 1C).

**Figure 2:**
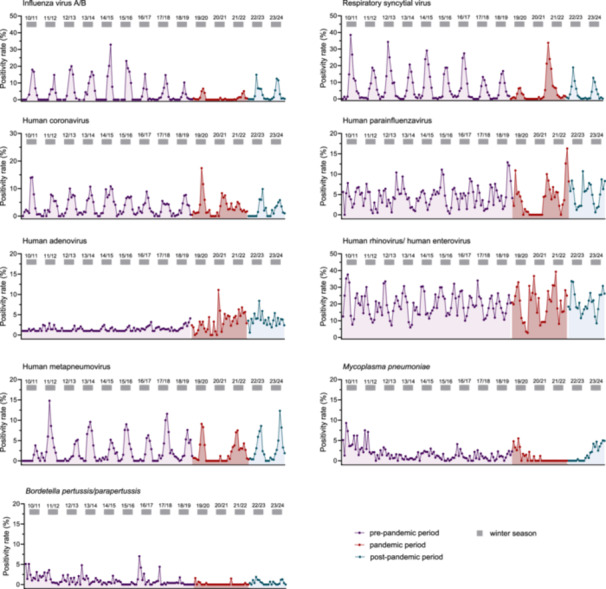
Activity and seasonality of respiratory viruses and atypical bacteria. Percentage of patients testing positive for any non‐SARS‐CoV‐2 respiratory virus or atypical bacterium among all patients tested by syndromic multiplex panel tests per month for the indicated year. Human rhinovirus and human enterovirus were reported as a combined target in multiplex panel testing. The gray bar indicates the respective winter season, spanning from November to February of the specified years.

**Figure 3:**
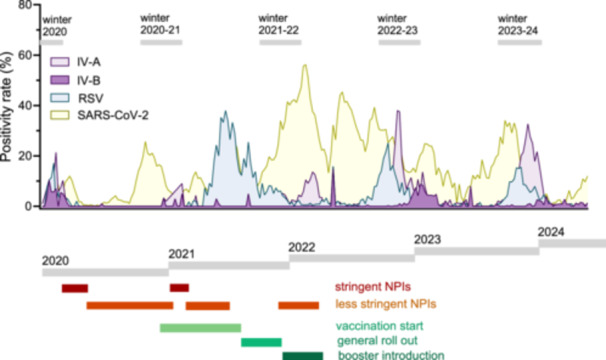
Influenza virus and respiratory syncytial virus detection following the emergence of SARS‐CoV‐2. Percentage of patients testing positive for SARS‐CoV‐2, influenza virus A, influenza virus B or respiratory syncytial virus among all patients tested by syndromic multiplex panel tests per surveillance week (from calendar week 1 in 2020 to calendar week 26 in 2024). In Switzerland, governmental non‐pharmaceutical interventions (NPIs) were introduced in calendar week 11 of 2020. Stringent NPIs included the prohibition of gatherings at private and public events, suspension of global travel, border closures to EU/EFTA countries, closure of non‐essential retail stores, mandatory remote work, mask mandates, and enforced isolation or quarantine for COVID‐19 cases. Less stringent NPIs involved mandatory mask‐wearing in enclosed public indoor spaces (such as public transport, offices, schools, etc.), physical distancing, and recommendations for remote work. For the full timeline of all NPIs, see Supplementary Table 1. Indication for SARS‐CoV‐2 testing changed over the study period and was conducted using different nucleic acid testing methods (Supplementary Table 5). SARS‐CoV‐2 vaccination is shown as green bars. The gray bar represents the respective winter season, spanning from November to February of the specified years.

During the pre‐pandemic periods, IV‐A/B, RSV, seasonal HCoVs, and human metapneumovirus (hMPV) consistently exhibited peak activity during the winter months each year. In contrast, human rhinovirus/human enterovirus (HRV/HEV) displayed biannual seasonal peaks, one in winter and one in summer, while human adenovirus (HAdV) infections were consistently detected throughout the year. Human parainfluenza virus (HPIV) infections were observed year‐round, with peak activity noted for HPIV‐1, HPIV‐2 and HPIV‐4 during the autumn and winter months, and for HPIV 3 during the spring and summer months (Figure 2; Supplementary Figure 1). The seasonal patterns of respiratory virus activity were significantly disrupted following the emergence of SARS‐CoV‐2 in Switzerland in February 2020. Although the first weeks of 2020 were dominated by respiratory viruses other than SARS‐CoV‐2, the emergence of SARS‐CoV‐2, followed by the implementation of NPIs led to an unprecedented decline in their activity (Figure 3). Within just 3 weeks, seasonally circulating respiratory viruses were almost entirely replaced by SARS‐CoV‐2 [3]. During the pandemic winter seasons, positivity rates for IV‐A, IV‐B, RSV, hMPV, HCoV, HPIV and atypical bacteria dropped substantially, leading to a 70–90% decline in their activity (Table 1). Specifically, IV‐A positivity rates dropped to 2–50% of pre‐pandemic levels, IV‐B to 3–30%, and RSV to 2–30% (Table 1). Notably, the decrease in detection of non‐SARS‐CoV‐2 respiratory pathogens coincided with ongoing SARS‐CoV‐2 circulation. SARS‐CoV‐2 cases initially peaked in March 2020, followed by significant surges during the winters of 2020–21 and 2021–22 (Figure 3). In contrast, HRV/HEV and HAdV seemed to be less impacted (Figure 2, Table 1). Their activity maintained an average positivity rate of 13.7% for HRV/HEV and 4.5% for HAdV during the pandemic 2020–21 season, compared to < 1% for most other respiratory pathogens (Table 1).

**Table 1:** Weekly test numbers and positivity rates for non‐SARS‐CoV‐2 respiratory viruses and atypical bacteria during the pandemic winter seasons (2019–20, 2020–2021, and 2021–22) compared to pre‐pandemic periods (9 winter seasons; 2010–11 to 2018–19).

Respiratory pathogen	Pre‐pandemic Period^a^		Pandemic‐ Period 2019 –20^b^			Pandemic‐ Period 2020–21^c^			Pandemic‐ Period 2021–22^d^		
	Weekly number of laboratory tests (mean; 95% CI)	Positivity rate (mean; 95% CI)	Weekly number of laboratory tests (mean; 95% CI)	Positivity rate (mean; 95% CI)	Rate ratio (mean; 95% CI)^e^	Weekly number of laboratory tests (mean; 95% CI)	Positivity rate (mean; 95% CI)	Rate ratio (mean; 95% CI)^e^	Weekly number of laboratory tests (mean; 95% CI)	Positivity rate (mean; 95% CI)	Rate ratio (mean; 95% CI)^e^
Human Adenovirus	118 (98–139)	1.4% (1.3–1.5%)	121 (82–160)	2.4% (1.6–3.2%)	1.7 (0.6–2.9)	62 (50–74)	4.5% (2.1–6.9%)	3.2 (0–6.1)	261 (229–293)	4.6% (3.9–5.3%)	3.3 (0.7–5.8)
Human Coronavirus (types 229E, HKU1, NL63, OC43)		5.7% (3.9–7.4%)		3.8% (1.3% to 6.2%)	0.7 (0.2–1.1)		3.3% (1.4–5.2%)	0.6 (0.3–0.9)		3.8% (3.1–4.5%)	0.7 (0.3–1.0)
Human Metapneumovirus		3.6% (2.5–4.6%)		4.1% (1.8–6.4%)	1.15 (0.6–1.7)		0.2% (0.0–0.5%)	0.07 (0.0–0.2)		4.6% (3.4–5.8%)	1.3 (0.8–1.8)
Human Parainfluenzavirus (types 1 to 4)		3.2% (2.0–4.3%)		3.4% (1.3–6.2%)	1.1 (0.7–1.4)		0.0% (0.0–0.0%)	0.0 (0.0–0.0)		3.3% (1.9–4.8%)	1.1 (0.7–1.4)
Human Rhinovirus/Enterovirus		19.6% (17.9–21.2%)		17.6% (12.3–22.8%)	0.9 (0.7–1.1)		13.7% (9.5–22.8%)	0.7 (0.5–0.9)		21.7% (14.3–29.1%)	1.1 (0.8–1.5)
Influenza virus A		13.9% (8.9–18.8%)		6.5% (4.1–8.9%)	0.5 (0.3–0.7)		0.3% (0.02–0.5%)	0.02 (0.0–0.04)		1.9% (1.1–2.6%)	0.13 (0.0–0.3)
Influenza virus B		8.9% (6.5–11.3%)		2.3% (0.9% to 3.6%)	0.3 (0.06 to 0.45)		0.3% (0.02–0.5%)	0.03 (0.0–0.09)		0.0% (0.0– 0.0%)	0.0 (0.0–0.0)
Respiratory syncytial virus		14.1% (9.2–18.9%)		4.3% (2.9–5.7%)	0.3 (0.2–0.4)		0.3% (0.02–0.5%)	0.02 (0.01–0.04)		4.3% (0.9–7.6%)	0.3 (0.0–0.7)
*Mycoplasma pneumoniae*		2.0% (1.6–2.4%)		2.7% (1.7% to 3.8%)	1.4 (0.9–1.9)		0.4% (0.05–0.7%)	0.19 (0.0–0.4)		0.0% (0.0–0.0%)	0.0 (0.0–0.0)
*Bordetella pertussis*		1.0% (0.6–1.4%)		0.1% (0–0.2%)	0.1 (0.0–0.3)		0.0% (0.0–0.0%)	0.0 (0.0–0.0)		0.0% (0.0–0.0%)	0.0 (0.0–0.0)

^a^ average of laboratory tests performed and proportion of patients with a positive non‐SARS‐CoV‐2 respiratory virus or bacterial detection over 9 winter seasons, from 1st November to 28th/29th February spanning the years 2010–11 to 2018–19.

^b^ weekly number of laboratory tests performed and proportion of patients with a positive non‐SARS‐CoV‐2 respiratory virus or bacterial detection from 1st November 2019 to 29th February 2020.

^c^ weekly number of laboratory tests performed and proportion of patients with a positive non‐SARS‐CoV‐2 respiratory virus or bacterial detection from 1st November 2020 to 28th February 2021.

^d^ weekly number of laboratory tests performed and proportion of patients with a positive non‐SARS‐CoV‐2 respiratory virus or bacterial detection from 1st November 2021 to 28th February 2022.

^e^ the rate ratio is calculated by comparing the proportion of patients with a positive non‐SARS‐CoV‐2 respiratory virus or bacterial detection in the indicated year to the pre‐pandemic level. A rate ratio of 1 indicates no difference in positivity rates between pandemic and pre‐pandemic seasons; > 1 indicates a higher positivity rate in pandemic compared to pre‐pandemic seasons; < 1 indicates a lower positivity rate in pandemic compared to pre‐pandemic seasons.

**Figure 4:**
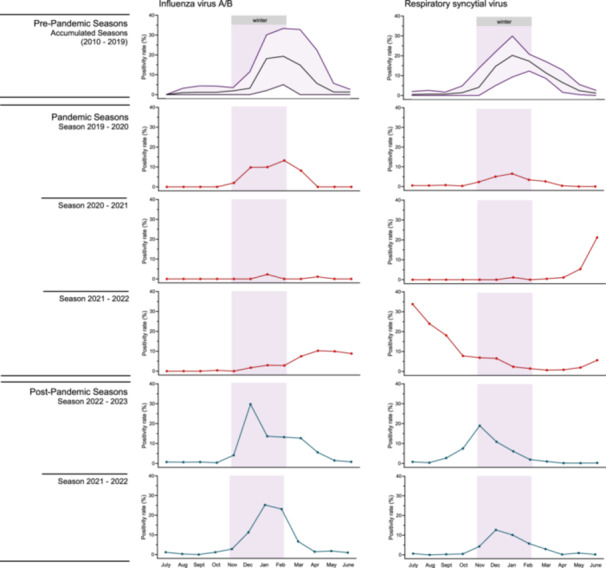
Activity and seasonality of influenza virus A/B and respiratory syncytial virus in pre‐pandemic, pandemic and post‐pandemic periods. Top panel: The purple line indicates the average positivity rate for influenza virus‐A/B or respiratory syncytial virus per month for the pre‐pandemic seasons (9 seasons, 2010–11 to 2018–19). The light purple shaded areas around each line represent the range of maximum and minimum positivity rates during the pre‐pandemic seasons. Middle and bottom panel: percentage of patients testing positive for influenza virus‐A/B or respiratory syncytial virus among all patients tested by syndromic multiplex panel tests per month for the indicated year, spanning from July 1st to June 30th of the indicated years. The dark purple bar represents the typical seasonal activity of influenza virus A/B or respiratory syncytial virus.

Based on these findings, we assessed shifts in peak activity of respiratory pathogens during the pandemic and subsequent post‐pandemic seasons. Compiled data on IV‐A/B epidemics during the 9 surveillance years preceding the COVID‐19 pandemic showed IV‐A/B activity typically beginning in November and peaking in January, with an offset until March (Figure 4). Following the emergence of SARS‐CoV‐2, IV‐A/B activity declined in March 2020, remained historically low throughout the summer of 2020, and was absent in the 2020–21 winter season. The 2021–22 influenza epidemic began late in January 2022,

peaked at a positivity rate of 10.2% in April, and persisted until June. In the following post‐pandemic seasons, seasonal patterns gradually re‐established, with IV‐A/B epidemics peaking at 29.8% in December 2022 and 25.2% in January 2024 (Figure 4, Supplementary Figure 1).

Cumulative pre‐pandemic RSV activity typically began in October, peaked in January, and subsided by March. During the pandemic winter seasons, RSV positivity rates dropped substantially. Following the easing of NPIs, an early epidemic onset occured in May 2021, peaking at 33.8% positivity in July and persisting until December (Figure 3, Figure 4). In the post‐pandemic winters of 2022–23 and 2023–24, RSV seasonality returned to pre‐pandemic patterns, with peak activity in November 2022 and December 2023

When assessing the activity of other respiratory viruses, including hMPV, HPIV types 1–4 and HCoV types –229E, –HKU1, –NL63, and –OC43, we observed similar patterns of significantly reduced positivity rates during the pandemic seasons. This was followed by substantial off‐season activity after the lifting of NPIs, and a subsequent return to pre‐pandemic seasonality in the post‐pandemic periods (Supplementary Figure 1). Notably, bacterial pathogens such as *M. pneumoniae* and *B. pertussis* were completely absent during pandemic years, but showed a strong resurge with increased positivity rates in the 2022–2023 and 2023–2024 post‐pandemic seasons (Figure 2, Table 1).

We next assessed whether the age distribution of patients with RTIs differed across study periods. In the pre‐pandemic period, 25,737 patients were tested (median age 60 years, IQR 27–76), of whom 27.0% were children. During the pandemic, 12,410 patients were tested (median age 59 years, IQR 29–75), with 23.1% pediatric patients. In the post‐pandemic period, 18,372 patients were tested (median age 62 years, IQR 36–76), with 20.4% children. Although statistical testing indicated significant differences in median patient age between periods (*p* < 0.001), this is likely driven by the large sample size analyzed (*n* = 56,519), as median values and interquartile ranges were highly similar. By contrast, the proportion of pediatric patients declined stepwise, from 27.0% pre‐pandemic to 23.1% during the pandemic and 20.4% post‐pandemic, indicating that children constituted a progressively smaller proportion of the tested patient population in the later years (Supplementary Table 4).

**Figure 5:**
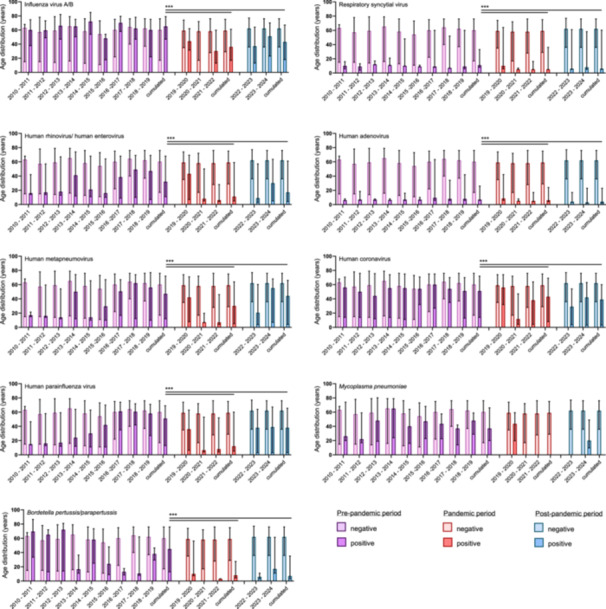
Age distribution of patients with respiratory tract infections. Age distribution of patients who tested positive or negative for non–SARS‐CoV‐2 respiratory pathogens or atypical bacteria during the pre‐pandemic (2010–11 to 2018–19; purple), pandemic (2019–20, 2020–21, and 2021–22; red), and post‐pandemic (2022–23 and 2023–24; blue) periods. Statistical testing was performed on the cumulated age distributions of patients testing positive for each respective respiratory pathogen across the study periods. Pairwise comparisons were conducted between the pre‐pandemic versus pandemic and the pre‐pandemic versus post‐pandemic periods. *p* values were calculated using the Mann–Whitney U test, applying Bonferroni correction for multiple comparisons. A single asterisk (*) denotes a significance level of *p* < 0.05, a double asterisk (**) for *p* < 0.01, and a triple asterisk (***) for *p* < 0.001.

Analyzing the age distribution of IV‐A/B‐positive cases (Figure 5), we found a cumulative median patient age of 65 years (IQR: 47–79 years) during the pre‐pandemic that significantly decreased to 36 years (IQR: 17–61 years) during the pandemic. This shift was likely driven by a higher impact on pediatric patients, as evidenced by the increased proportion of IV‐A/B cases among patients ≤ 18 years, reaching 31.2% in the 2021–22 winter season, following a complete absence of viral activity during the 2020–21 period (Figure 5; Supplementary Figure 2). In the post‐pandemic 2023–24 season, the median patient age returned to pre‐pandemic levels at 51 years (IQR: 23–70 years), with a decrease of affected pediatric patients to 21.2% (Figure 5; Supplementary Figure 2). Similar trends were observed for HCoV and HPIV infections, with significantly younger patients during the COVID‐19 pandemic (*p* < 0.001; Figure 5) and a significantly higher proportion of pediatric patients affected (*p* < 0.001; Supplementary Figure 2). After the complete absence of RSV circulation during the pandemic, RSV activity resurged following the lifting of NPIs, leading to an off‐season RSV epidemic during the 2020–21 season. This resurgence disproportionately affected pediatric patients, who accounted for a significantly increased 75.1% of cases. However, this proportion decreased to 58% in subsequent seasons, with an increase in RSV infections among adult patients. Correspondingly, the median patient age dropped to 5 years (IQR: 5–36 years) during the pandemic but reverted to 8 years (IQR: 5–58 years) in the post‐pandemic 2023–24 season, approaching the pre‐pandemic median of 11 years (IQR: 8–33 years) (Figure 5; Supplementary Figure 2). For HRV/HEV, we observed that pediatric and adult patients were alternately more affected during the pre‐pandemic periods, as evidenced by shifts in the median patient age and the proportions of pediatric and adult patients affected (Figure 5; Supplementary Figure 2). During the 2020–21 and 2021–22 seasons, a high proportion of up to 71% of pediatric patients were affected, accompanied by a significant decrease in the median patient age. A similar trend was observed for hMPV, while HAdV infections consistently predominated among pediatric patients across all assessed periods. For *M. pneumoniae*, the median patient age was 37 years (IQR: 20–66 years) during the pre‐pandemic. *M. pneumoniae* activity was absent following the emergence of SARS‐CoV‐2 in 2020; however, a surge in cases was observed in the 2023–24 post‐pandemic season, with a median patient age of 20 years and a significantly higher proportion of pediatric patients affected (*p* < 0.001; Figure 5; Supplementary Figure 2). Similarily, paediatric patients were primarily affected when *B. pertussis/B. parapertussis* infections resurged in the 2022–23 season, after its absence during pandemic years. The following 2023–24 post‐pandemic season saw a return to higher median patient ages (Figure 5; Supplementary Figure 2).

**Figure 6:**
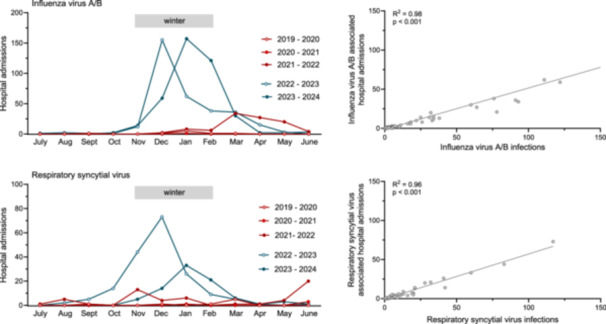
Influenza virus A/B and respiratory syncytial virus associated hospitalizations. Left panel: Monthly number of hospital admissions for adult patients aged > 18 years associated with influenza virus A/B or respiratory syncytial virus infections during the pandemic (2019–20, 2020–21, 2021–22; red) and post‐pandemic periods (2022–23, 2023–24; blue). Right panel: Linear regression analysis of the number of influenza virus A/B or respiratory syncytial virus infections and associated hospital admissions among adult patients aged > 18 years during the pandemic (2019–20, 2020–21, 2021–22; red) and post‐pandemic periods (2022–23, 2023–24; blue). Hospitalizations are defined as admissions in patients presenting with acute RTIs at the emergency or outpatient clinics who tested positive for influenza virus A/B or RSV and were subsequently hospitalized within 24 h.

Finally, we assessed hospital admissions among adult patients across pandemic and post‐pandemic seasons. SARS‐CoV‐2 emerged during the typical influenza season in Europe, coinciding with a decline in hospital admissions during the 2019–20 season. No IV‐A/B‐associated hospitalizations were recorded in the 2020–21 season, while off‐season admissions occurred between February and April 2022. In the post‐pandemic period, increased IV‐A/B activity and higher case numbers were associated with a rise in hospital admissions temporally linked to these infections (R² > 0.98; *p* < 0.001; Figure 6), peaking at 155 and 157 cases in December and January of the 2022–23 and 2023–24 seasons, respectively (Figure 6). A similar trend was observed for RSV, with off‐season hospitalizations peaking in June 2022, followed by a significant increase in RSV infections and related hospital admissions during the 2022–23 season (R^2^ > 0.96; *p* < 0.001; Figure 6).

## 4 Discussion

This comprehensive study of the viral and bacterial etiologies of RTIs in pediatric and adult populations in northwestern Switzerland across 14 years, including pre‐pandemic, pandemic, and post‐pandemic periods, showed significant shifts in the etiological landscape of RTIs, providing valuable insights into their evolving epidemiology and clinical impact.

Before the COVID‐19 pandemic, respiratory viruses such as IV‐A/B, RSV, HCoV and HPIV followed distinct seasonal patterns, peaking in winter, while HRV and HAdV circulated throughout the year [2], with their relative prevalence decreasing in winter due to interference with other viruses like IV‐A/B [16]. The emergence of SARS‐CoV‐2 and the implementation of NPIs in early 2020 led to a near‐complete displacement of seasonally circulating respiratory viruses within just 3 weeks [3, 4, 5]. Surveillance data revealed a significant increase in SARS‐CoV‐2 infections, alongside a marked decline in IV‐A/B and RSV cases across various climates [6, 7, 17, 18]. Other non‐SARS‐CoV‐2 viruses also exhibited altered circulation patterns. Seasonal HCoV [6, 17, 19], HPIV [6, 20], and hMPV [6, 20] declined sharply at the onset of the pandemic, coinciding with the implementation of stringent NPIs. The reduction in respiratory virus circulation was most pronounced during the initial phase of the COVID‐19 pandemic when most stringent NPIs were enforced and persisted to varying degrees during subsequent waves of SARS‐CoV‐2 when novel variants emerged [21, 22].

Notably, non‐enveloped viruses like HRV and HAdV were less affected by NPIs. Although both declined at the pandemic′s onset [6, 17], they rebounded faster than other respiratory viruses. This suggests that their environmental stability, ability to persist on surfaces, and transmission through both direct contact and fomites make them less susceptible to NPIs, though their transmission dynamics are also shaped by shifts in population immunity and the timing of NPIs [23]. Furthermore, their extensive genomic diversity with > 100 distinct serotypes limits cross‐neutralization, leaving individuals susceptible to reinfection by different strains. Indeed, HRV infections were consistently detected in both pediatric and adult populations, with shifts between age groups across different seasons, likely reflecting the circulation of distinct HRV types that elicit different immune responses [24]. Our internal validation further supports that HRV/HEV detections in our data set represent HRV circulation in the vast majority of cases ( > 90%; data not shown), consistent with previous reports that respiratory syndromic multiplex panel tests predominantly detect rhinoviruses rather than enteroviruses [8].

By mid‐2022, governments eased COVID‐19 restrictions. With the potential waning of population immunity due to reduced virus exposure during the pandemic years, and fewer preventive measures in place, there was a subsequent surge in respiratory virus circulation. Our study observed an unusual inter‐seasonal surge in RSV infections and hospitalizations in 2021, mirroring a global trend, in which RSV activity peaked in winter instead of summer in Australia and South Africa [7, 25, 26], and in summer‐fall instead of winter in Europe, the U.S. and Canada [27, 28]. These findings suggest that the easing of NPIs, along with a rising “immunity debt” in the population after prolonged periods without exposure to certain viruses, contributed to these atypical seasonal trends [29]. In 2022, the RSV season started earlier than usual in the Northern Hemisphere, and RSV‐associated hospitalizations peaked between October and December [30, 31]. Similarly, IV‐A/B resurgence was a major concern following NPI relaxation. Our data align with global trends showing a later occurrence of the 2021–22 influenza season in the Northern Hemisphere as compared to pre‐pandemic years, with shorter seasons in Canada and Europe and a longer season in the U.S [32, 33]. By winter 2022–23, IV‐A/B activity rebounded with an unusually early onset in Europe, the U.S., and Canada [34, 35, 36].

Our findings also show shifts in the typical age distribution of RTIs. RSV is known to primarily affect young children, with the highest incidence in infants. However, in our tertiary care hospital that primarily serves adults, a significant number of individuals over 65 years were also affected. IV‐A/B was more prevalent among older adults, while HCoV, HPIV, and HRV/HEV infections remained common across all age groups. During the second and third year of the pandemic, pediatric RTIs due to IV‐A/B, RSV, HCoV, and HPIV surged. In the post‐pandemic period, infections shifted towards older children and adults, reflecting a delayed exposure‐driven susceptibility. Reduced exposure to respiratory viruses early in the COVID‐19 pandemic likely contributed to waning population immunity as levels of antivirus specific antibodies declined [37], leading to an “immunity debt” [29]. In the first months of life, infants are partially protected by maternal antibodies transferred transplacentally. Lower levels of maternal antibodies being passed to infants during pregnancy may have resulted in a birth cohort of immunologically naive infants or infants with low levels of maternal antibodies, making them more vulnerable during their first viral exposure. This may have facilitated viral transmission among pediatric populations after the easing of NPIs when schools and childcare facilities reopened, leading to secondary household transmission affecting high‐risk groups such as older adults. Immunologically naive children or those with limited prior exposure to RSV during the COVID‐19 pandemic may have experienced more severe disease with a higher risk of hospitalization as compared to pre‐pandemic years [38, 39]. Our study confirms that adult populations were also affected by consecutive major RSV epidemics in the 2022–2023 and 2023–2024 seasons, with increased RSV‐associated hospitalizations as compared to pre‐pandemic periods.

Similar trends were observed for IV‐A/B, with a notable increase in viral activity among paediatric populations during the early COVID‐19 pandemic after NPIs were eased, while post‐pandemic seasons showed a return to pre‐pandemic patterns [40, 41]. Early in the pandemic, IV‐A/B‐associated hospitalizations dropped significantly, and remained at historically low levels until the 2021–22 influenza season. This aligns with surveillance data from Switzerland [42], other European countries [43], the U.S. [32], Canada [6], Australia [20], and South Africa [7], all of which reported declines in IV‐A/B‐associated hospitalizations and mortality. By 2021–22, IV‐A/B‐associated hospitalizations returned to pre‐pandemic levels [44]. The subsequent 2022–23 and 2023–24 seasons saw hospitalization rates surpassing pre‐pandemic levels, consistent with reports from North America, Europe and Australia [32, 33, 34, 35, 40, 41]. This surge likely reflects a combination of increased virus circulation, waning population immunity, reduced vaccine coverage, and antigenic drift of circulating strains, underscoring the urgent need for enhanced influenza surveillance and vaccine adaptation [41, 45].

Vaccination dynamics during the study period likely contributed to respiratory pathogen circulation and disease burden. Influenza vaccination uptake rose temporarily by about 5–10% in 2020–21, particularly among older adults, supported by pandemic‐related campaigns, but declined again in 2023–24 by 3–10% compared with the two preceding seasons in both the United States and Europe [46, 47, 48]. This transient rise in influenza vaccine uptake during the 2020–21 season, together with NPIs, likely contributed to historically low influenza incidence during the first pandemic winter, whereas reduced coverage in later years may have contributed to the high case numbers and associated hospitalizations observed after 2022.

Pertussis remains an important respiratory pathogen, particularly in young children, although vaccination uptake in high‐income countries has generally been high. In the United States and Europe, infant coverage has consistently exceeded 90% over the past decade and remained above this level through 2023–24 [49, 50]. Complementary strategies have further strengthened protection. Maternal immunization, recommended in many European countries for the past 5–12 years, has been associated with significant reductions in hospitalizations among infants [51]. In contrast, uptake of adolescent and adult boosters has remained limited [52, 53]. In our epidemiological study, pertussis activity was absent in 2020–21 but re‐emerged in 2023–24 at levels comparable to the pre‐pandemic period, consistent with U.S. and European surveillance reports [49, 54]. This resurgence likely reflects an “immunity gap” resulting from reduced pathogen circulation during COVID‐19 restrictions, which limited opportunities for natural boosting of immunity. Waning vaccine‐induced protection and persistently low uptake of adolescent and adult boosters may have further contributed to the observed rebound [55, 56]. Comparable dynamics were observed for other atypical bacteria. *Mycoplasma pneumoniae* incidence declined markedly during the COVID‐19 pandemic, reaching its lowest incidence in over a decade [57, 58]. Following the relaxation of NPIs, cases and associated hospitalizations increased, consistent with the concept of an “immunity gap” caused by reduced exposure during the pandemic, which likely contributed to heightened post‐pandemic susceptibility.

At the same time, COVID‐19 vaccination markedly reduced the burden of severe courses of COVID‐19, altering the relationship between infection incidence and clinical outcomes. In Switzerland, vaccination began in late December 2020 with prioritization of healthcare workers, older adults, and other high‐risk groups. By mid‐2021, eligibility was expanded to the general adult population, and by the end of 2021 approximately 67% of the population was fully vaccinated. Comparable coverage levels were reported in the United States and Europe [59, 60]. The rapid uptake among high‐risk groups was associated with a decoupling of infection incidence from severe outcomes, as hospitalization and mortality rates were substantially lower among fully vaccinated individuals, particularly those who had received booster doses, compared with unvaccinated populations [61, 62]. The widespread rollout of vaccines, together with the policy‐driven relaxation of NPIs in 2022, created the conditions for the re‐emergence of other respiratory viruses, reflecting both restored contact patterns and altered population immunity after 2 years of suppressed circulation. In addition, viral interference with widespread SARS‐CoV‐2 circulation during the early pandemic waves likely further suppressed the activity of other respiratory viruses, including influenza and RSV [3].

Taken together, recent vaccination dynamics, including temporary increases followed by subsequent declines in influenza vaccination coverage among older adults, sustained high infant pertussis coverage with persistent gaps in adolescent and adult booster uptake, and the widespread rollout of COVID‐19 vaccines, likely contributed to the post‐2022 shifts in susceptibility and disease burden observed across multiple respiratory pathogens in the United States and Europe. These trends underscore the complex interplay of vaccination, waning and cohort‐specific immunity, viral interference, and behavioral changes such as mobility patterns and the resumption of social contact, indicating that vaccination alone does not fully account for the observed epidemiological changes.

This study leveraging comprehensive epidemiological data from extensive molecular syndromic multiplex panel testing over a 14‐year period at two major tertiary care hospitals in northwestern Switzerland represents one of the longest continuous surveillance periods in comparable research, and allowed us to assess long‐term trends in respiratory pathogen dynamics. Following the significant impacts of SARS‐CoV‐2 interference alongside the implementation of NPIs on respiratory virus circulation, post‐pandemic seasons reveal a notable resurgence of seasonal patterns. However, this ‘return to normal′ is accompanied by a hgher disease burden, and altered age distributions, indicating that a full restortion of pre‐pandemic age ditributions patterns and RTI incidence levels may require ongoing viral circulation to rebuild population immunity. Several factors, including differences in the virulence of epidemic IV‐A/B and RSV strains and advancements in RSV prophylaxis, such as the monoclonal antibody Nirsevimab® (Sanofi and AstraZeneca) [63], and newly approved vaccines Arexvy® (GSK) [64], and Abrysvo® (Pfizer) [65] may also influence these trends. Given the uncertainties around post‐pandemic viral circulation, our findings emphasize the critical need for ongoing surveillance of respiratory virus activity and seasonal trends for refining vaccination strategies for vaccine‐preventable RTIs. These adaptive public health strategies are essential to anticipate and mitigate future respiratory virus surges in pediatric and older populations, who may be more vulnerable due to potential immunity gaps following the COVID‐19 pandemic, and for reducing overall disease burden.

### 4.1 Limitations

This study has some limitations. First, syndromic multiplex testing platforms varied over the study period. Given the high sensitivity of nucleic acid testing in symptomatic patients [3, 5, 9, 10], these methodological differences are unlikely to have significantly affected detection rates. Second, variations in testing regimens during the pandemic may have influenced virus detection; though, testing behavior alone rarely explains the magnitude of observed infection waves [66]. Third, reluctance to seek medical care early in the pandemic may have led to underreporting of RTIs. Since other viral illnesses remained low even after COVID‐19 restrictions were lifted, healthcare avoidance alone may not fully explain decreased notifications, and emergency physicians involved in this study did not note a major decline in patient numbers during the pandemic. Fourth, pandemic‐driven behavioral and procedural changes may have influenced sampling practices. Although the clinical indication for multiplex panel testing remained unchanged during the whole study period and was restricted to patients presenting with clinically relevant RTI symptoms, heightened public awareness during the pandemic may have resulted in some patients with milder illness presenting for hospital care. This may have contributed to the observed surge in testing volumes in early 2020 and the sustained higher testing thereafter. While statistical analyses indicated significant differences in patient age distribution across the pre‐pandemic, pandemic, and post‐pandemic years (Supplementary Table 4), these are likely attributable to the very large sample size analyzed (*n* = 56,519). Median ages (59–62 years) and interquartile ranges, as well as the proportions of pediatric patients (20–27%), were highly similar across periods, indicating that the overall study population remained demographically stable. This stability provides important context, as the substantial shifts observed in the median age of patients with specific respiratory pathogen detections can therefore be interpreted as true epidemiological changes rather than artifacts of differences in the underlying study population. Fifth, we did not perform medical chart reviews to adjudicate whether hospitalizations were directly attributable to IV‐A/B or RSV. Instead, we applied strict criteria, defining hospitalization as the admission of a patient who presented to the emergency department or outpatient clinics with acute RTI symptoms, tested positive by multiplex panel test, and was admitted to the hospital within 24 h of testing. While our study provides valuable insights into the rebounds of respiratory virus circulation patterns and aligns with current surveillance data [40, 41, 42], these limitations emphasize the need to consider potential confounding factors when interpreting long‐term epidemiological virus data.

## 5 Conclusion

Our analysis of patients with RTIs over nine pre‐pandemic, three pandemic, and two post‐pandemic seasons highlights the profound impacts of SARS‐CoV‐2 emergence and NPIs on the epidemiology of respiratory viruses and bacteria, including shifts in etiology, seasonality, age distribution, disease burden, and related hospitalizations. While the first pandemic year caused a dramatic change in the etiology of RTIs, the subsequent easing of public health measures led to the resurgence of multiple respiratory pathogens, albeit with persistent age‐related disparities. After the pandemic, virus‐specific seasonality is gradually returning, with patterns starting to resemble those seen pre‐pandemic. However, shifts in affected age groups and fluctuations in virus circulation indicate that full epidemiological normalization has yet to occur. This underscores the need for continued surveillance and adaptive public health strategies to mitigate future disruptions in respiratory virus dynamics.

## Author Contributions

Rainer Gosert, Sarah Tschudin‐Sutter, and Karoline Leuzinger contributed to the conceptualization and study design. Data collection and clinical contributions were provided by Klaudia Naegele, Roland Bingisser, Christian H. Nickel, Jakob Meyer, Martin Siegemund, Stefano Bassetti, Sarah Dräger, Christoph T. Berger, Fabian Franzek, OP, Ulrich Heininger, Julia Bielicki, AE, Hans H. Hirsch, MW, Nina Khanna, Sarah Tschudin‐Sutter. Methodology and laboratory analyses were conducted by Rainer Gosert, Klaudia Naegele, Peter M. Keller, AE, Hans H. Hirsch and Karoline Leuzinger. Data analysis and interpretation were performed by Rainer Gosert, MW, Klaudia Naegele, Peter M. Keller, Sarah Tschudin‐Sutter, and Karoline Leuzinger. The original draft of the manuscript was written by Rainer Gosert and Karoline Leuzinger. All authors reviewed and approved the final manuscript.

## Ethics Statement

The study was conducted in accordance with Good Laboratory Practice, the Declaration of Helsinki, and all applicable national and institutional guidelines. It was approved by the local ethics committee (EKNZ 2020‐00769).

## Conflicts of Interest

The authors declare no conflicts of interest.

## Supporting information


**Supplementary Figure 1:** Activity and seasonality of respiratory viruses and bacteria in pre‐pandemic, pandemic and post‐pandemic periods.

**Supplementary Figure 2:** Proportion of pediatric patients among respiratory pathogen positive cases.

**Supplementary Table 1:** Timeline of non‐pharmaceutical interventions implemented in Switzerland.

**Supplementary Table 2:** Viral and bacterial targets of the respiratory syndromic multiplex panel tests.

**Supplementary Table 3:** Patient demographics.

**Supplementary Table 4:** Patient demographics over the pre‐pandemic (9 seasons, 2010–11 to 2018–19), pandemic (2019–20, 2020–21, and 2021–22), and post‐pandemic (2022–23 and 2023–24) periods.

**Supplementary Table 4:** Patient demographics over the pre‐pandemic (9 seasons, 2010–11 to 2018–19), pandemic (2019–20, 2020–21, and 2021–22), and post‐pandemic (2022–23 and 2023–24) periods.

## Data Availability

The data that support the findings of this study are available from the corresponding author upon reasonable request.
