# Supplementary material for: Rebound of Respiratory Virus Activity and Seasonality to Pre‐Pandemic Patterns
Source: J Med Virol. 2025 Oct 23;97(11):e70658. doi: 10.1002/jmv.70658 (PMC12548497; doi:10.1002/jmv.70658)
Supplement: Supplementary file 1 — Supplementary Figure 1: Activity and seasonality of respiratory viruses and bacteria in pre‐pandemic, pandemic and post‐pandemic periods. [file JMV-97-e70658-s005.pdf]

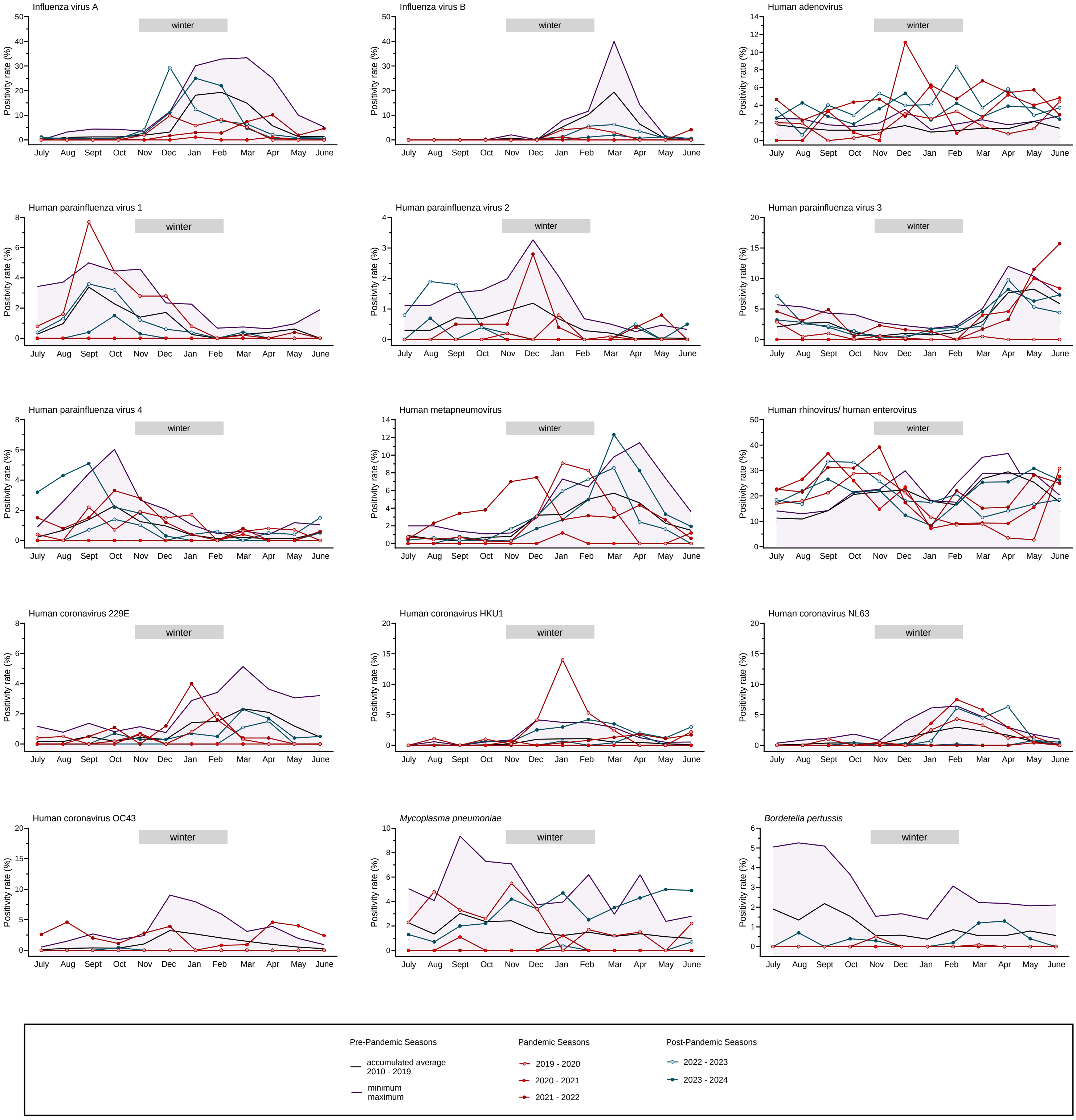

Supplementary Figure 1. Activity and seasonality of respiratory viruses and bacteria in pre-pandemic, pandemic and post-pandemic periods.
The dark purple line represents the average percentage of patients testing positive for the respective respiratory pathogen among all patients tested by syndromic multiplex panel tests per month for the baseline seasons (9 seasons, 2010–11 to 2018–19). The shaded area indicates the range of maximum and minimum positivity rates during the pre-pandemic seasons. The red and blue lines indicate the percentage of patients testing positive for the respective respiratory pathogen among all patients tested by syndromic multiplex panel tests during the specified pandemic and post-pandemic seasons, spanning from July 1st to June 30th of the indicated year.
