# Supplementary material for: Rebound of Respiratory Virus Activity and Seasonality to Pre‐Pandemic Patterns
Source: J Med Virol. 2025 Oct 23;97(11):e70658. doi: 10.1002/jmv.70658 (PMC12548497; doi:10.1002/jmv.70658)
Supplement: Supplementary file 2 — Supplementary Figure 2: Proportion of pediatric patients among respiratory pathogen positive cases. [file JMV-97-e70658-s006.pdf]

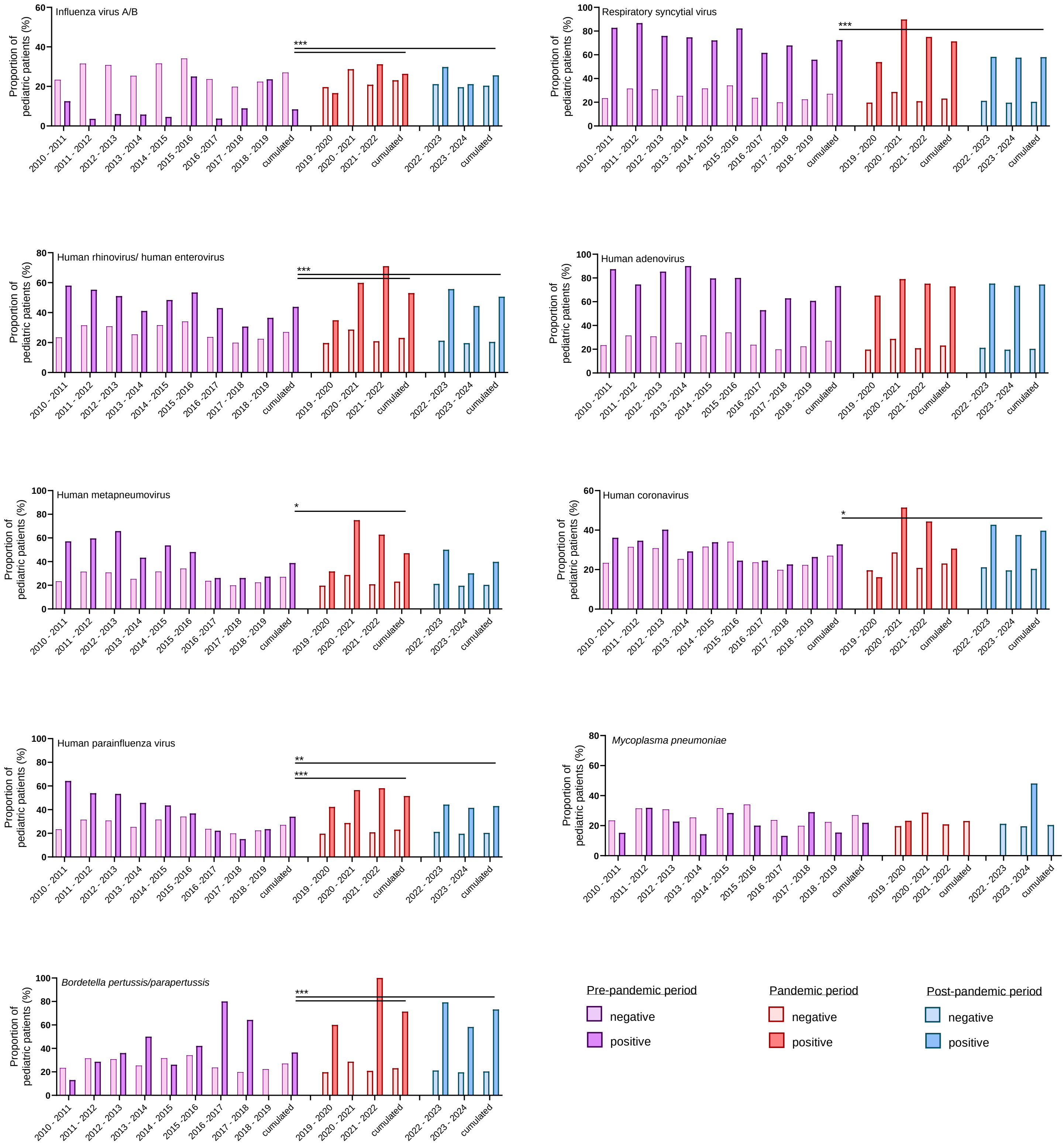

Supplementary Figure 2. Proportion of pediatric patients among respiratory pathogen positive cases

Proportion of pediatric patients among respiratory pathogen negative and positive cases across the pre-pandemic (2020–11 to 2018–19; purple), pandemic (2019–20, 2020–21, and 2021–22; red), and post-pandemic (2022–23 and 2023–24; blue) periods. P-values indicate significant differences in cumulated age distributions between the pre-pandemic, pandemic, and post-pandemic periods, respectively. P-values were calculated using the Mann Whitney U test, with a single asterisk (*) denoting a significance level of p <0.05, a double asterisk (**) for p <0.01, and a triple asterisk (***) for p <0.001.
