# Supplementary material for: Rebound of Respiratory Virus Activity and Seasonality to Pre‐Pandemic Patterns
Source: J Med Virol. 2025 Oct 23;97(11):e70658. doi: 10.1002/jmv.70658 (PMC12548497; doi:10.1002/jmv.70658)
Supplement: Supplementary file 3 — Supplementary Table 1: Timeline of non‐pharmaceutical interventions implemented in Switzerland. [file JMV-97-e70658-s003.docx]

**Supplementary Table 1.** Timeline of non-pharmaceutical interventions implemented in Switzerland

| **Date Calendar week**  **Year** | **Phase** | **Intervention** |
| --- | --- | --- |
| 28.02.2020  calendar week-9  2020 | Emergence of SARS-CoV-2 in Switzerland | Restrictions were placed on mass gatherings, with large events of more than 1’000 people prohibited. For events with fewer than 1’000 participants, the cantonal authorities were required to conduct a risk assessment. |
| 13.03.2020  calendar week-11  2020 | Introduction of non-pharmaceutical interventions | The Federal Council banned events with more than 100 people until the end of April and a maximum of 50 people were allowed to stay in restaurants, bars and discos. Face-to-face teaching in schools was banned until April 4^th^. |
| 16.03.2020  calendar week-12  2020 | First Lockdown (16.03.2020 – 10.05.2020) | All shops (except groceries), markets, restaurants, bars and entertainment and leisure establishments remained closed and there was a ban on private and public events. Switzerland introduced border controls and entry restrictions to its neighboring states, with the exception of the Principality of Liechtenstein. |
| 19.03.2020  calendar week-12  2020 |  | Entry restrictions for travelers from outside the Schengen area. |
| 20.03.2020  calendar week-12  2020 |  | Gatherings of more than five people were prohibited. |
| 27.04.2020  calendar week-18  2020 | Stepwise lifting of non-pharmaceutical interventions  Phase I  (11.05.2020 – 18.10.2020) | Hairdressers, cosmetic studios, hardware stores, flower shops and garden centers were allowed to reopen. Hospitals were allowed to conduct all medical procedures again. |
| 11.05.2020  calendar week-20  2020 | Stepwise lifting of non-pharmaceutical interventions  Phase II  (11.05.2020 – 18.10.2020) | Shops, restaurants, markets, museums, and libraries were allowed to reopen. Lessons in primary and secondary schools were allowed to take place on site again. The entry regulations at the borders were relaxed for EU and EFTA citizens. |
| 30.05.2020  calendar week-22  2020 | Stepwise lifting of non-pharmaceutical interventions  Phase III  (11.05.2020 – 18.10.2020) | The ban on gatherings was relaxed. |
| 06.06.2020  calendar week-23  2020 | Stepwise lifting of non-pharmaceutical interventions  Phase IV  (11.05.2020 – 18.10.2020) | Private and public events with up to 300 people were permitted again (e. g. family events, trade fairs, concerts, theater performances or film screenings). |
| 15.06.2020  calendar week-25  2020 | Stepwise lifting of non-pharmaceutical interventions  Phase V  (11.05.2020 – 18.10.2020) | The borders to all countries within the EU/EFTA area were fully reopened. |
| 20.06.2020  calendar week-25  2020 | Stepwise lifting of non-pharmaceutical interventions  Phase VI  (11.05.2020 – 18.10.2020) | Events with up to 1’000 people were possible again. The home office recommendation was lifted. Obligation to wear a mask for people aged twelve and older on all public transport remained in place. |
| 19.10.2020  calendar week-43  2020 | Reinstatement of non-pharmaceutical interventions  (19.10.2020 – 17.01.2021) | Requirement to wear a mask was extended to all enclosed public indoor spaces, including train platforms, bus stops, airports, restaurants and hotels. |
| 28.10.2020  calendar week-44  2020 |  | Requirement to wear a mask outdoors, the ban on private meetings with more than ten people and the closure of bars and discos. |
| 02.11.2020  calendar week-45  2020 |  | Universities were required to transition to remote learning, while in-person instruction continued to be allowed in compulsory education and high schools. |
| 23.12.2020  calendar week-52  2020 |  | Vaccination campaign started in Switzerland, prioritizing healthcare workers, older adults, and other high-risk groups. |
| 19.01.2021  calendar week-3  2021 | Second Lockdown (18.01.2021 – 27.02.2021) | Re-enforcement of protective NPIs came into force again. Among others all shops (except groceries), markets, restaurants, bars and entertainment and leisure establishments remained closed. |
| 01.03.2021  calendar week-9  2021 | Relaxation of non-pharmaceutical interventions  (01.03.2021 – 07.09.2021) | Shops, museums and libraries were allowed to reopen, as well as outdoor areas of sports and leisure facilities, zoos, and botanical gardens. Meetings with family and friends as well as sporting and cultural activities with up to 15 people were permitted again outdoors. |
| 22.03.2021  calendar week-12  2021 |  | The restriction on indoor meetings with family and friends was relaxed from five to a maximum of ten people. |
| 31.05.2021  calendar week-22  2021 |  | Restaurants were allowed to reopen their indoor areas with protective concepts in place. There were also relaxations for sporting and cultural events. Thermal baths and wellness facilities were allowed to reopen. |
| 26.06.2021  calendar week-25  2021 |  | NPIs are being greatly reduced and simplified. Among others, the home office requirement and the mask requirement outdoors was lifted. A general mask mandate remains in effect for indoor areas.  Vaccination eligibility expanded to all adults ≥16 years; coverage increased rapidly over summer. |
| 08.09.2021  calendar week-36  2021 |  | A “COVID certificate requirement” was put in place. A certificate of either SARS-CoV-2 vaccination or negative SARS-CoV-2 testing was required upon entry to restaurants, cultural and leisure facilities and at indoor events. |
| 20.09.2021  calendar week-38  2021 |  | Individuals who were neither vaccinated nor recovered from SARS-CoV-2 infection had to present a negative antigen or PCR test upon entry to Switzerland. Additionally, a follow-up test conducted in Switzerland was required four to seven days later. |
| 06.12.2021  calendar week-49  2021 | Reinstatement of non-pharmaceutical interventions  (06.12.2021 – 01.02.2022) | Stricter NPIs came in effect throughout Switzerland. The mask and certificate requirements were expanded. In addition, companies, restaurants, bars and event organizers were allowed to introduce a 2G rule (SARS-CoV-2 vaccinated or recovered from SARS-CoV-2 infection). In addition, an urgent home office recommendation was put in place. |
| 20.12.2021  calendar week-51  2021 |  | Federal Council was expanding the 2G rule. It applied to all indoor events as well as the interiors of restaurants, cultural, sports and leisure establishments.  By the end of 2021, approximately 67% of the Swiss population was fully vaccinated; comparable uptake was achieved in neighboring European countries. |
| 13.01.2022  calendar week-2  2022 |  | Shorter quarantine and extension of the 2G rule: People without symptoms could be exempt from corona isolation or quarantine after just five days. |
| 03.02.2022  calendar week-5  2022 | Relaxation of non-pharmaceutical interventions | The Federal Council was lifting the home office requirement and the contact quarantine. |
| 17.02.2022  calendar week-7  2022 |  | The Federal Council was largely cancelling the coronavirus protection measures. Certificates and masks were no longer compulsory in shops, restaurants and cultural venues. The mask requirement on public transport and the five-day isolation period for SARS-CoV-2 infected persons remained in place. |
| 01.04.2022  calendar week-13  2022 | Lifting of all non-pharmaceutical interventions | The isolation requirement for infected people, the mask requirement on public transport, and in health care facilities was lifted. |
