# Supplementary material for: Rebound of Respiratory Virus Activity and Seasonality to Pre‐Pandemic Patterns
Source: J Med Virol. 2025 Oct 23;97(11):e70658. doi: 10.1002/jmv.70658 (PMC12548497; doi:10.1002/jmv.70658)
Supplement: Supplementary file 4 — Supplementary Table 2: Viral and bacterial targets of the respiratory syndromic multiplex panel tests. [file JMV-97-e70658-s007.docx]

**Supplementary Table 2.** Viral and bacterial targets of the respiratory syndromic multiplex panel tests.

| Respiratory Panel | RespiFinder-22®  Pathogen Panel | Luminex® NxTAG Respiratory Pathogen Panel | BioFire® Respiratory  Pathogen Panel | Xpert® XpressCoV-2/Flu/RSV plus |
| --- | --- | --- | --- | --- |
| Time RP used | July 2010 –  January 2016 | January 2016 –  March 2017 | November 2016  to date | October 2021  to date |
| Company | PathoFinder  (Maastricht, The Netherlands) | Diasorin  (Saluggia, Italy) | bioMérieux  (Marcy-l’Étoile, France) | Cepheid  (CA, USA) |
| Viral targets covered | Adenovirus | Adenovirus | Adenovirus | - |
|  | Human coronavirus 229E | Human coronavirus 229E | Human coronavirus 229E | - |
|  | Human coronavirus HKU1 | Human coronavirus HKU1 | Human coronavirus HKU1 | - |
|  | Human coronavirus NL63 | Human coronavirus NL63 | Human coronavirus NL63 | - |
|  | Human coronavirus OC43 | Human coronavirus OC43 | Human coronavirus OC43 | - |
|  | Human metapneumovirus | Human metapneumovirus | Human metapneumovirus | - |
|  | Influenza A virus | Influenza A virus | Influenza A virus | Influenza A virus |
|  | Influenza A/H3 | Influenza A/H3 | Influenza A/H3 |  |
|  | Influenza A/H1 | Influenza A/H1 | Influenza A/H1 |  |
|  | - | - | Influenza A/H1-2009 |  |
|  | Influenza B virus | Influenza B virus | Influenza B virus | Influenza B virus |
|  | Parainfluenza virus 1 | Parainfluenza virus 1 | Parainfluenza virus 1 | - |
|  | Parainfluenza virus 2 | Parainfluenza virus 2 | Parainfluenza virus 2 | - |
|  | Parainfluenza virus 3 | Parainfluenza virus 3 | Parainfluenza virus 3 | - |
|  | Parainfluenza virus 4 | Parainfluenza virus 4 | Parainfluenza virus 4 | - |
|  | Respiratory syncytial virus A | Respiratory syncytial virus A | Respiratory syncytial virus A+B | Respiratory syncytial virus A+B |
|  | Respiratory syncytial virus B | Respiratory syncytial virus B |  |  |
|  | Human Rhinovirus | Human Rhinovirus/Enterovirus | Human Rhinovirus/Enterovirus | - |
|  | Human Bocavirus | Human Bocavirus | - | - |
|  | - | - | SARS-CoV-2  (since version 2.1) | SARS-CoV-2 |
| Bacterial targets covered | *Mycoplasma pneumoniae* | *Mycoplasma pneumoniae* | *Mycoplasma pneumoniae* | - |
|  | *Bordetella pertussis* | *Bordetella pertussis*  *LDT ^1^* | *Bordetella pertussis* | *-* |
|  | *Bordetella parapertussis*  *LDT^1^* | *Bordetella parapertussis*  *LDT ^1^* | *Bordetella parapertussis* | *-* |
|  | *Legionella pneumophila* | *-* | *-* |  |
|  | *Chlamydophila pneumoniae* | *Chlamydophila pneumoniae* | *Chlamydophila pneumoniae* |  |
| Number of targets | *22* | *20* | *22* | 4 |
| Time to result | 8 – 16h | ~4h | ~1h | ~1h |

^1^ Since these targets were not included in the syndromic multiplex panel testing systems, they were tested concurrently using a laboratory-developed test.
