# Supplementary material for: Rebound of Respiratory Virus Activity and Seasonality to Pre‐Pandemic Patterns
Source: J Med Virol. 2025 Oct 23;97(11):e70658. doi: 10.1002/jmv.70658 (PMC12548497; doi:10.1002/jmv.70658)
Supplement: Supplementary file 5 — Supplementary Table 3: Patient demographics. [file JMV-97-e70658-s002.docx]

**Supplementary Table 3.** Patient demographics

| Patients (n) |  | 56’519 |
| --- | --- | --- |
|  | Female patients (n, %) | 26’382 (46.7%) |
|  | Pediatric patients ≤18 years (n, %) | 13’187 (23.3%) |
|  | Age adult patients (years) | Median: 66  25^th^ P: 50  75^th^ P: 75  Min: 19  Max: 111 |
|  | Age pediatric patients (years) | Median: 6  25^th^ P: 3  75^th^ P: 10  Min: 1  Max: 18 |
| Samples (n) |  | 83’823 |
|  | Nasopharyngeal swab (n, %) | 73’286 (87.4%) |
|  | Bronchoalveolar Lavage (n, %) | 6’955 (8.3%) |
|  | Nasal swab (n, %) | 1’966 (2.3%) |
|  | Throat swab (n, %) | 864 (1.1%) |
|  | Tracheal/bronchial secretion (n, %) | 512 (0.6%) |
|  | Sputum (n, %) | 240 (0.3%) |
