# Supplementary material for: Rebound of Respiratory Virus Activity and Seasonality to Pre‐Pandemic Patterns
Source: J Med Virol. 2025 Oct 23;97(11):e70658. doi: 10.1002/jmv.70658 (PMC12548497; doi:10.1002/jmv.70658)
Supplement: Supplementary file 6 — Supplementary Table 4: Patient demographics over the pre‐pandemic (9 seasons, 2010–11 to 2018–19), pandemic (2019–20, 2020–21, and 2021–22), and post‐pandemic (2022–23 and 2023–24) periods. [file JMV-97-e70658-s001.docx]

**Supplementary Table 4.** Patient demographics over the pre-pandemic (9 seasons, 2010–11 to 2018–19), pandemic (2019–20, 2020–21, and 2021–22), and post-pandemic (2022–23 and 2023–24) periods.

| Time period | Patients (n) |  | 2’092 |
| --- | --- | --- | --- |
| 2010–11 |  | Female patients (n, %) | 986 (47.1%) |
| (July 1^st^ 2010 |  | Pediatric patients ≤18 years (n, %) | 490 (23.4%) |
| to June 30^th^ 2011) |  | Patient age (years) | Median: 63  25^th^ P: 15  75^th^ P: 69  Min: 1  Max: 106 |
| Time period | Patients (n) |  | 2’537 |
| 2011–12 |  | Female patients (n, %) | 1’131 (44.6%) |
| (July 1^st^ 2011 |  | Pediatric patients ≤18 years (n, %) | 800 (31.5%) |
| to June 30^th^ 2012) |  | Patient age (years) | Median: 57  25^th^ P: 15  75^th^ P: 78  Min: 1  Max: 103 |
| Time period | Patients (n) |  | 3’340 |
| 2012–13 |  | Female patients (n, %) | 1’529 (45.8%) |
| (July 1^st^ 2017 |  | Pediatric patients ≤18 years (n, %) | 1’029 (30.8%) |
| to June 30^th^ 2018) |  | Patient age (years) | Median: 60  25^th^ P: 14  75^th^ P: 79  Min: 1  Max: 105 |
| Time period | Patients (n) |  | 2’869 |
| 2013–14 |  | Female patients (n, %) | 1’196 (41.7%) |
| (July 1^st^ 2013 |  | Pediatric patients ≤18 years (n, %) | 730 (25.4%) |
| to June 30^th^ 2014) |  | Patient age (years) | Median: 66  25^th^ P: 18  75^th^ P: 79  Min: 1  Max: 111 |
| Time period | Patients (n) |  | 1’946 |
| 2014–15 |  | Female patients (n, %) | 856 (44.0%) |
| (July 1^st^ 2014 |  | Pediatric patients ≤18 years (n, %) | 616 (31.7%) |
| to June 30^th^ 2015) |  | Patient age (years) | Median: 58  25^th^ P: 13  75^th^ P: 76  Min: 1  Max: 107 |
| Time period | Patients (n) |  | 1’991 |
| 2015–16 |  | Female patients (n, %) | 883 (44.3%) |
| (July 1^st^ 2015 |  | Pediatric patients ≤18 years (n, %) | 680 (34.2%) |
| to June 30^th^ 2016) |  | Patient age (years) | Median: 54  25^th^ P: 11  75^th^ P: 73  Min: 1  Max: 104 |
| Time period | Patients (n) |  | 2’982 |
| 2016–17 |  | Female patients (n, %) | 1’337 (44.8%) |
| (July 1^st^ 2016 |  | Pediatric patients ≤18 years (n, %) | 707 (23.7%) |
| to June 30^th^ 2017) |  | Patient age (years) | Median: 60  25^th^ P: 23  75^th^ P: 75  Min: 1  Max: 106 |
|  |  |  |  |
| Time period | Patients (n) |  | 3’533 |
| 2017–18 |  | Female patients (n, %) | 1’721 (48.7%) |
| (July 1^st^ 2017 |  | Pediatric patients ≤18 years (n, %) | 703 (19.9%) |
| to June 30^th^ 2018) |  | Patient age (years) | Median: 64  25^th^ P: 40  75^th^ P: 76  Min: 1  Max: 105 |
| Time period | Patients (n) |  | 4’447 |
| 2018–19 |  | Female patients (n, %) | 2’130 (47.9%) |
| (July 1^st^ 2018 |  | Pediatric patients ≤18 years (n, %) | 996 (22.4%) |
| to June 30^th^ 2019) |  | Patient age (years) | Median: 62  25^th^ P: 37  75^th^ P: 76  Min: 1  Max: 106 |
| Time period | Patients (n) |  | 3’387 |
| 2019–20 |  | Female patients (n, %) | 1’589 (46.9%) |
| (July 1^st^ 2019 |  | Pediatric patients ≤18 years (n, %) | 668 (19.7%) |
| to June 30^th^ 2020) |  | Patient age (years) | Median: 59  25^th^ P: 35  75^th^ P: 74  Min: 1  Max: 106 |
| Time period | Patients (n) |  | 1’679 |
| 2020–21 |  | Female patients (n, %) | 809 (48.2%) |
| (July 1^st^ 2020 |  | Pediatric patients ≤18 years (n, %) | 482 (28.7%) |
| to June 30^th^ 2021) |  | Patient age (years) | Median: 58  25^th^ P: 17  75^th^ P: 72  Min: 1  Max: 100 |
| Time period | Patients (n) |  | 7’344 |
| 2021–22 |  | Female patients (n, %) | 3’474 (47.3%) |
| (July 1^st^ 2021 |  | Pediatric patients ≤18 years (n, %) | 1’535 (20.9%) |
| to June 30^th^ 2022) |  | Patient age (years) | Median: 58  25^th^ P: 28  75^th^ P: 76  Min: 1  Max: 105 |
| Time period | Patients (n) |  | 9’465 |
| 2022–23 |  | Female patients (n, %) | 4’572 (48.3%) |
| (July 1^st^ 2022 |  | Pediatric patients ≤18 years (n, %) | 2’007 (21.2%) |
| to June 30^th^ 2023) |  | Patient age (years) | Median: 62  25^th^ P: 36  75^th^ P: 77  Min: 1  Max: 103 |
| Time period | Patients (n) |  | 8’907 |
| 2023–24 |  | Female patients (n, %) | 4’169 (46.8%) |
| (July 1^st^ 2023 |  | Pediatric patients ≤18 years (n, %) | 1’744 (19.6%) |
| to June 30^th^ 2024) |  | Patient age (years) | Median: 62  25^th^ P: 36  75^th^ P: 76  Min: 1  Max: 105 |
|  |  |  |  |
| Cumulated | Patients (n) |  | 25’737 |
| pre-pandemic period |  | Female patients (n, %) | 11’695 (45.4%) |
| (July 1^st^ 2010 |  | Pediatric patients ≤18 years (n, %) | 6’949 (27.0%) |
| to June 30^th^ 2019) |  | Patient age (years) | Median: 60  25^th^ P: 27  75^th^ P: 76  Min: 1  Max: 111 |
| Cumulated | Patients (n) |  | 12’410 |
| pandemic period |  | Female patients (n, %) | 5’891 (47.5%) |
| (July 1^st^ 2019 |  | Pediatric patients ≤18 years (n, %) | 2’869 (23.1%) |
| to June 30^th^ 2022) |  | Patient age (years) | Median: 59  25^th^ P: 29  75^th^ P: 75  Min: 1  Max: 106 |
| Cumulated | Patients (n) |  | 18’372 |
| post-pandemic period |  | Female patients (n, %) | 8’737 (47.6%) |
| (July 1^st^ 2022 |  | Pediatric patients ≤18 years (n, %) | 3’746 (20.4%) |
| to June 30^th^ 2024) |  | Patient age (years) | Median: 62  25^th^ P: 36  75^th^ P: 76  Min: 1  Max: 105 |
