# Supplementary material for: Rebound of Respiratory Virus Activity and Seasonality to Pre‐Pandemic Patterns
Source: J Med Virol. 2025 Oct 23;97(11):e70658. doi: 10.1002/jmv.70658 (PMC12548497; doi:10.1002/jmv.70658)
Supplement: Supplementary file 7 — Supplementary Table 4: Patient demographics over the pre‐pandemic (9 seasons, 2010–11 to 2018–19), pandemic (2019–20, 2020–21, and 2021–22), and post‐pandemic (2022–23 and 2023–24) periods. [file JMV-97-e70658-s004.docx]

**Supplementary Table 5.** Timeline of SARS-CoV-2 screening strategies for asymptomatic patients implemented at the University Hospital Basel during the COVID-19 pandemic.

| Year | Month | SARS-CoV-2 PCR Screening | | Screening Strategy for Asymptomatic Patients |
| --- | --- | --- | --- | --- |
| 2020 | 1 |  |  |  |
| 2020 | 2 | SARS-CoV-2 LDT [3] |  |  |
| 2020 | 3 |  |  | Systematic swabs by the mobile screening team (pre-hospital admission and pre-operative) 30.03.20 - 15.06.20 |
| 2020 | 4 | cobas ® SARS-CoV-2 (Roche, Rotkreuz, Switzerland) [5] |  |  |
| 2020 | 5 |  |  |  |
| 2020 | 6 |  |  |  |
| 2020 | 7 |  |  | No systematic screening |
| 2020 | 8 |  |  |  |
| 2020 | 9 |  |  |  |
| 2020 | 10 |  |  |  |
| 2020 | 11 |  |  |  |
| 2020 | 12 |  |  |  |
| 2021 | 1 |  |  | Systematic swabs by the mobile screening team once per week 14.01.21 - 08.02.21 |
| 2021 | 2 |  |  |  |
| 2021 | 3 |  |  | Systematic sputum screening 08.02.21 - 06.07.21   Screening started on 08.02.21 at Day 0 and Day 7  Screening strategy was adjusted on 16.03.21 to Day 0 and Day 5 |
| 2021 | 4 |  |  |  |
| 2021 | 5 |  |  |  |
| 2021 | 6 |  |  |  |
| 2021 | 7 |  |  |  |
| 2021 | 8 |  |  | Systematic sputum screening 25.08.21 - 05.12.22   Screening started on 25.08.21 at Day 0 and Day 5  Screening strategy was adjusted from 18.03.22 on to Day 0, Day 3 and Day 5 |
| 2021 | 9 |  |  |  |
| 2021 | 10 |  | Xpert® Xpress SARS-CoV-2  (Cepheid, CA, USA) [9] |  |
| 2021 | 11 |  |  |  |
| 2021 | 12 |  |  |  |
| 2022 | 1 |  |  |  |
| 2022 | 2 |  |  |  |
| 2022 | 3 |  |  |  |
| 2022 | 4 |  |  |  |
| 2022 | 5 |  |  |  |
| 2022 | 6 |  |  |  |
| 2022 | 7 |  |  |  |
| 2022 | 8 |  |  |  |
| 2022 | 9 |  |  |  |
| 2022 | 10 |  |  |  |
| 2022 | 11 |  |  |  |
| 2022 | 12 |  |  |  |
| 2023 | 1 |  |  | No systematic screening |
| 2023 | 2 |  |  |  |
| 2023 | 3 |  |  |  |
| 2023 | 4 |  |  |  |
| 2023 | 5 |  |  |  |
| 2023 | 6 |  |  |  |
| 2023 | 7 |  |  |  |
| 2023 | 8 |  |  |  |
| 2023 | 9 |  |  |  |
| 2023 | 10 |  |  |  |
| 2023 | 11 |  |  |  |
| 2023 | 12 |  |  |  |
| 2024 | 1 |  |  |  |
| 2024 | 2 |  |  |  |
| 2024 | 3 |  |  |  |
| 2024 | 4 |  |  |  |
| 2024 | 5 |  |  |  |
| 2024 | 6 |  |  |  |
